# Validity of a food frequency questionnaire to assess nutritional intake among Sri Lankan adults

**DOI:** 10.1186/s40064-016-1837-x

**Published:** 2016-02-24

**Authors:** Ranil Jayawardena, Nuala M. Byrne, Mario J. Soares, Prasad Katulanda, Andrew P. Hills

**Affiliations:** Department of Physiology, Faculty of Medicine, University of Colombo, Colombo, Sri Lanka; Institute of Health and Biomedical Innovation, Faculty of Health, Queensland University of Technology, Brisbane, QLD Australia; Bond Institute of Health and Sport, Bond University, Gold Coast, Australia; Curtin Health Innovation Research Institute, School of Public Health, Faculty of Health Sciences, Curtin University, Perth, WA Australia; Diabetes Research Unit, Faculty of Medicine, University of Colombo, Colombo, Sri Lanka; School of Health Sciences, University of Tasmania, Launceston, TAS Australia

**Keywords:** FFQ, Sri Lanka, Adults, Validation, Diet, Nutrition

## Abstract

Sri Lanka is undergoing nutritional transition and diet-related chronic diseases are emerging as an important health problem. Currently, no validated food frequency questionnaire (FFQ) exists to measure habitual dietary intake of Sri Lankan adults. The purpose of the study is to assess the validity of a semi-quantitative FFQ and 7-day weighed-intake dietary records (7DWR), designed to assess dietary intake among Sri Lankan adults. Dietary intake was measured using both a FFQ and 7DWR. The FFQ consisted of 8 food groups containing the main foods comprising the diet of Sri Lankan adults, a total of 85 items and 12 color photographs to identify serving size. One hundred healthy adults were randomly recruited from a community sample and administrated the FFQ followed by completion of the 7DWR. Paired sample t tests, Pearson’s correlation coefficients, kappa test and Bland–Altman analysis were conducted to determine correlation and the level of agreement for energy and micronutrients. Seventy-seven participants completed both the FFQ and 7DWR. Estimated mean energy intake (SD) from FFQ (1794 ± 398 kcal) and 7DWR (1698 ± 333 kcal, p < 0.001) were significantly different due to a significant overestimation of carbohydrate (11.5 g/day, p < 0.001) and to some extent fat (5.7 g/day, ns). Significant positive correlations (p < 0.05) were found between the FFQ and 7DWR for energy (r = 0.39), carbohydrate (r = 0.47), protein (r = 0.26), fat (r = 0.17) and dietary fiber (r = 0.32). Bland–Altman graphs indicated fairly good agreement between methods with no relationship between bias and average intake of each nutrient examined. Based on these findings, the FFQ appears to be an acceptable tool for assessing the nutrient intake of Sri Lankans and will assist proper categorization of individuals by dietary intake.

**Electronic supplementary material:**

The online version of this article (doi:10.1186/s40064-016-1837-x) contains supplementary material, which is available to authorized users.

## Introduction

Sri Lanka is a low-middle-income country (LMIC) undergoing a nutritional transition (Jayawardena et al. 2014). Although under-nutrition and anemia are still prevalent, a significant proportion of adults are suffering from diet-associated non-communicable diseases (NCDs; Jayawardena et al. 2014). For example, a quarter of Sri Lankan adults are suffering from the metabolic syndrome (Katulanda et al. 2012) and using the Sri Lankan classification of obesity (BMI > 25 kg m^−2^), 21 % men and 33 % women are obese (Jayawardena et al. 2012a). Dietary pattern plays a significant role in obesity (Jayawardena et al. 2013) and an estimated one-fifth of adults are dysglycemic (Katulanda et al. 2008). A priority area for Sri Lankan health authorities is to combat diet-related NCDs however there is limited information on dietary patterns to supplement national data on diabetes and cardiovascular diseases, mainly due to the absence of a valid nutrition assessment tool (Katulanda et al. 2008).

FFQs are designed to measure dietary intake over an extended period of time, and is a commonly accepted tool to assess habitual dietary intake in epidemiological studies of diet and chronic diseases (Willett et al. 1988). In comparison to other dietary intake assessment methods, FFQs are relatively inexpensive, easy and quick to administer (Cade et al. 2002). In the US, a FFQ has been used to examine the relationship between fruit and vegetable intake and cardiovascular disease risk (Bazzano et al. 2002). Similarly, validated FFQs have been used for the European Prospective Investigation into Cancer and Nutrition (EPIC) Study in several countries (Kroke et al. 1999), and to obtain dietary data from a large adult sample in the Australian National Nutrition Survey (Mishra et al. 2002). FFQ is a valid tool for the assessment of dietary habits of South Indians (Dwarkanath et al. 2012).

Population-specific FFQs are important to assess the dietary intake of particular groups of people (Katulanda et al. 2008), including a multi-ethnic population characteristic of Sri Lanka (Jayawardena et al. 2013). We developed a semi-quantitative FFQ for Sri Lankan adults and collected a representative study sample (n = 600) from across the island (Jayawardena et al. 2012b). The relative validity of FFQs is usually assessed by comparing their findings with a reference method however such an approach is usually time consuming, detailed and with high subject burden. Despite the lack of a universally accepted ‘reference method’ 7-day weighed-intake dietary records (7DWR) are widely considered the ‘gold standard’ approach to assess habitual diet (Swan 2004). The aim of this study was to assess the validity of a newly developed FFQ to estimate nutrient intake compared to 7DWR among Sri Lankan adults.

## Methods

### Background

In the Sri Lanka Diabetes and Cardiovascular Study (SLDCS) a multi-stage random-cluster sampling method was used to select a nationally representative sample (n = 5000) of non-institutionalized adults aged ≥18 years (Katulanda et al. 2008). A sub-sample of the SLDCS was used to develop a representative FFQ for Sri Lankan adults details of which have been published elsewhere (Jayawardena et al. 2012c). Ethical approval for the study was obtained from the Ethical Review Committee, University of Colombo, Sri Lanka and written informed consent was obtained from each participant before data collection.

### Study sample

A total of 100 adults were randomly selected to participate in the validation study from the SLDCS and stratified based on ethnicity and area of residence. Ten clusters were selected to represent urban and rural areas, and main ethnicities and each had ten participants. Participants adhering to a prescribed therapeutic diet or on a weight reduction diet were excluded from the study. Of the 100 adults initially selected, 18 individuals failed to complete the 7DWR. A further five were excluded before statistical analyses were undertaken as dietary records were NOT representative of habitual intake with very low (<800 kcal/day) and very high reported average food intake (>4000 kcal/day; Jayawardena et al. 2014).

### Dietary assessment

#### Food frequency questionnaire

The FFQ was developed from a representative sample the details of which have been published previously (Jayawardena et al. 2012b). In summary, The FFQ contains color photographs of three different portion sizes of four commonly consumed foods (rice, a green vegetable curry, lentil curry and chicken) and a list of food items (n = 85) with their portion sizes and frequencies. Each respondent reported consumption of each food according to (1) frequency per day; (2) frequency per week; or (3) frequency per month. Food items were categorized into eight food groups namely (1) cereals; (2) vegetables; (3) pulses; (4) meat; (5) fruits; (6) drinks; (7) miscellaneous; and (8) alcohol. The FFQ was interviewer-administered in the local language (Sinhalese and Tamil) by two investigators. The length of interview ranged from 15 to 20 min during which participants were asked to recall their usual portion size and intake of foods listed within the FFQ over the past month. Participants were then instructed to complete a seven-day weighed food record.

#### Seven-day weighed intake

Participants were advised to keep a weighed record of all food items and beverages consumed, both in and out of the home, over a period of seven consecutive days. Investigators provided verbal instructions and demonstration on site and daily telephone instructions were also provided for any specific queries. All particiapnts received a calibrated kitchen scale (Tanita KD-407) and a ‘Home Record’ diary to weigh home-cooked foods, and a smaller pocket-sized ‘Eating and Drinking Away From Home’ diary (the ‘Eating Out’ diary) for recording food intake when foods could not be weighed, generally foods eaten away from home. Unweighed food items were quantified by estimating size based on participant’s description however items were very limited such as biscuits and other bakery items.

#### Analysis

Energy and nutrient intakes were calculated using NutriSurvey 2007 (EBISpro, Germany), nutrient analysis software modified for Sri Lankan food items and recipes.

Initially, mean and SDs of energy, macronutrient and micronutrient intakes were determined for both FFQ and 7DWR. Differences and ratios between mean values obtained for each method were then calculated and Paired t test used to determine statistical significance of the difference. Correlation between the intake amounts for each method was evaluated by Pearson correlation analysis.

Data from 23 participants who could not complete both methods accurately were excluded from statistical analysis. To assess agreement between the FFQ and reference methods and to detect any bias, differences were plotted against the means, as suggested by Bland and Altman (1986). Minitab version 15.0 was used for statistical analysis and a p value <0.05 was considered statistically significant.

## Results

Participants were selected from different ethnic backgrounds and area of residence and a total of 77 (of 100) participants completed both 7DWR and FFQ accurately. Mean (SD) age was 46.5 (8.3) years, and average BMI was 23.8 kg m^−2^ (4.7). Sixty-five (84.4 %) were women and the majority were Sinhalese (n = 69). Thirty-eight participants were from a rural area, 31 from urban areas and eight from estates (tea and rubber plantation areas).

Mean (SD) energy intake from 7DWR was 1697.9 (333.3) kcal/day and corresponding values from FFQ was significantly higher (p < 0.05) at 1794.1 (397.6) kcal/day. In both methods, over two-thirds of energy was derived from carbohydrates, and fat provided 19.8 and 22.1 % of energy from 7DWR and FFQ, respectively. Only 12.3 % of energy was derived from protein in the 7DWR and the corresponding value for FFQ was 11.1 % (Table 1).

**Table 1:** Pearson correlation coefficient between mean daily intakes of nutrients estimated by 7DWR and an FFQ

Energy and nutrient (n = 77)	Intake from 7-day diet diary		FFQ		r value	p value
	Mean	SD	Mean	SD		
Energy (kcal/d)	1697.9	333.3	1794.1	397.6	0.39	<0.001
Protein (g)	53.4	12.9	50.1	11.0	0.26	0.02
Fat (g)	39.4	9.9	46.1	12.9	0.17	0.14
Carbohydrate (g)	292.4	65.9	303.9	75.7	0.47	<0.001
Dietary fiber (g)	14.1	5.4	21.8	9.4	0.32	0.005
PUFA (g)	2.7	1.4	2.4	1.8	0.37	<0.001
Cholesterol (mg)	15.8	25.0	9.4	7.9	0.23	0.05
Vitamin A (μg)	426.3	172.5	652.3	292.6	0.17	0.19
Vitamin E (mg)	1.9	1.4	2.6	1.3	0.09	0.46
Vitamin B_1_ (mg)	1.6	0.5	1.5	0.4	0.26	0.02
Vitamin B_2_ (mg)	1.2	0.6	1.2	0.4	0.25	0.03
Vitamin B_6_ (mg)	1.2	0.6	1.3	1.0	0.36	0.001
Folic acid (mg)	39.0	16.5	45.5	21.7	0.46	<0.001
Vitamin D (μg)	7.2	7.6	7.6	7.8	0.29	0.01
Vitamin C (mg)	33.9	21.4	67.9	42.3	0.21	0.07
Potassium (mg)	1765.5	484.6	1963.2	577.2	0.26	0.02
Calcium (mg)	540.7	145.2	677.4	229.4	0.33	0.004
Magnesium (mg)	258.0	100.2	308.9	124.7	0.46	<0.001
Phosphorus (mg)	1020.3	225.3	1107.4	292.2	0.36	0.002
Sodium (mg)	1812.7	790.2	1834.3	856.1	0.16	0.17
Iron (mg)	16.7	9.2	19.7	9.7	0.25	0.03
Zinc (mg)	7.1	1.8	7.3	2.3	0.12	0.28
% Energy from fat^a^	19.8	3.9	22.1	4.1	0.34	0.002
% Energy from protein^a^	12.3	2.3	11.1	1.4	0.52	0.0001
% Energy from carbohydrate^a^	67.6	5.1	66.7	4.8	0.40	0.0001

^a^Percentage of energy deriving from nutrients were calculated by below equation (energy from a given nutrient/total energy intake × 100)

The correlation between FFQ and 7DWR was 0.39 for energy intake (p < 0.001). Percentage of energy from fat (r = 0.34), protein (r = 0.52) and carbohydrates (r = 0.40) were significantly (p < 0.05) correlated between methods with Pearson’s correlation coefficients for protein r = 0.26 (p = 0.023), r = 0.47 for carbohydrate intake (p < 0.0001), and r = 0.17 for fat intake (p = ns). Dietary fiber (r = 0.32) and PUFA (r = 0.37) were significantly correlated (p < 0.005) between two methods, and a lower correlation was obtained for dietary cholesterol (r = 0.23; p = 0.05). Among vitamins assessed, five out of eight showed significant correlations (Vitamin B1, B2, B6, D and folic acid) whereas among seven minerals (K, Ca, Mg, P, Na, Fe and Zn), only sodium (r = 0.17) and zinc (r = 0.12) were not significantly correlated.

**Fig. 1:**
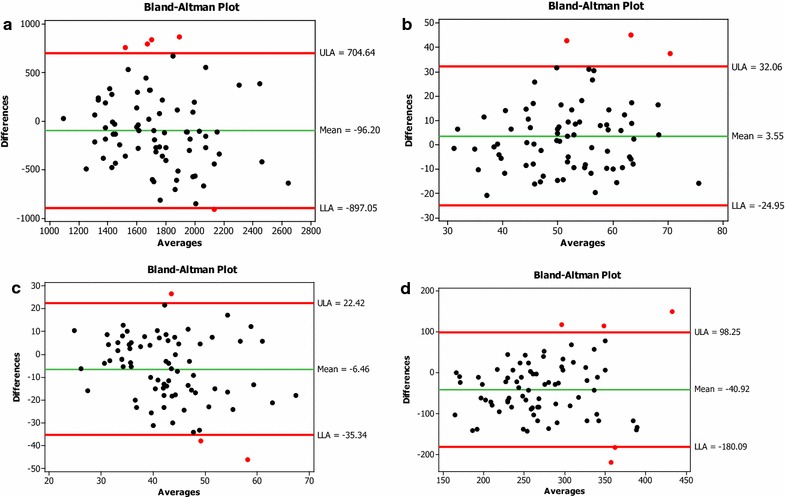
Bland and Altman plots for **a** energy (kcal), **b** protein in grams, **c** fat in grams, **d** carbohydrates in grams with the mean difference and limits of agreements. Averages = FFQ + &DWR/2. Mean difference (FFQ − 7DWR) is *green line* and 95 % limits of agreements in *red line*. *Red color dots* are outliers

The Bland and Altman plot (Fig. 1a), illustrates there was no tendency for energy differences between 7DWR and FFQ as absolute energy intake increased. Red dots represent outliers (Fig. 1). FFQ results indicated higher mean energy values compared to the reference method however the spread around the mean reflected consistent variation across all levels of intake. Similar to energy, carbohydrates, protein and fat, a few participants fell outside the limit of agreements (LOA). For all measurements, mean differences were not associated with the means of the two methods, confirming an acceptable level of agreement however LOA were wide (>±2SDs of the 7DWR) indicating poor agreement between FFQ and 7DWR across the range of intakes. In contrast, although fat and protein showed low correlation, LOA were well within ±2SDs.

## Discussion

Despite the high prevalence of diet-related illness in Sri Lanka, there is a lack of representative dietary data and its association with NCDs making it difficult to initiate effective preventive and curative strategies (Jayawardena et al. 2014). The main objective of the current study was to examine the validity of our FFQ to enable its application at the next national level NCD survey.

The 7DWR is considered the ‘gold standard’ method but is associated with considerable participant burden (Dwarkanath et al. 2012). Work commitments, either in the office or working in the fields places a distinct burden on the weighing of all food eaten during a particular day. Not surprisingly, many men refused to participate or discontinued the study and this may have limited the representativeness of the study population. Hence, our validation sample was mostly comprised of women (84.4 %). By comparison, the National Diet and Nutrition Survey—UK had a response rate for the 7DWR of less than 50 % (Swan 2004), significantly lower than in the current study.

The very high intake of starchy foods reported in a small study among Sri Lankan adults may be related to the epidemic of diabetes in the country (Jayawardena et al. 2012c). Approximately, 70 % of energy consumed by Sri Lankans is derived from starchy products (Jayawardena et al. 2014). Our FFQ showed a reasonable agreement in ranking of participants for intake of energy, macronutrients and some micronutrients compared to the reference method using both correlation analysis and quintile categories. Correlation coefficients for intake measured by the FFQ and 7DWR for energy and carbohydrate were 0.39 and 0.47, respectively. Although fat intake is also associated with many NCDs, it is practically difficult to measure in the Sri Lankan setting. There were also practical limitations in the administration of our food frequency questionnaire. Coconut oil is the main source of fat in Sri Lanka with almost all fats coming indirectly from vegetable and other cooked dishes. In such a situation a FFQ may not be the best method to estimate fat intake and additional questions regarding consumption of coconut milk and cooking oils provide a more complete picture. Similarly, it is difficult to quantify salt consumption in Sri Lankan due to varied cooking practices.

Bland–Altman is the preferred approach to assess agreement between FFQ and reference methods across a range of intakes (Cade et al. 2002). There were no systematic differences between FFQ and 7DWR for low or high intake of macronutrients.

In a European study, FFQ displayed correlation coefficients for nutrients compared to dietary recall ranging 0.54–0.86 (Kroke et al. 1999). A large epidemiological study to assess the risk factors of cancer (the JACC study) reported a correlation coefficient of 0.2 for energy between a 12-day weight diet record and FFQ (Date et al. 2005). A FFQ validated against multiple 24 h recalls in a south Indian population showed that correlation coefficients ranged from 0.11 for vitamin A to 0.44 for protein intake (Iqbal et al. 2009). Unlike Western diets, Asian meals are mainly comprised of mixed dishes and low correlations are expected. Our FFQ showed a satisfactory correlation for energy and major nutrients indicating suitability for application in studies on diet-related NCDs. However, there was wide variability in correlation in our study (0.52–0.17). Significant correlations for several micronutrients such as iron, calcium and vitamin D provide the possibility for use in studies on iron deficiency and osteoporosis in Sri Lanka. A significant proportion of Sri Lankans live abroad and this may contribute to a higher risk for diet-associated NCDs consistent with other south Asians. This FFQ may be a valid tool to assess nutrient intake of migrant Sri Lankans living in developed countries.

A limitation that should be acknowledged is the lack of information on the reproducibility (reliability) of the FFQ. Reproducibility is usually assessed by administering the questionnaire at two (or more) points in time to the same group of people. As the FFQ is designed to capture long-term intake, a judgment regarding appropriate time interval is difficult. However we did re-administer the same FFQ (FFQ2) 7–10 days after the first and we found a significant correlation between the two FFQs (Additional file 1: Table S1). We accept that accuracy may have been compromised to some extent as respondents may have remembered some of their previous responses (Cade et al. 2002). Finally, this FFQ is limited to measurement of food consumption across the previous month. Data regarding longer-term dietary habits are very important to better understand diet and associated diseases.

## Conclusion

Validation of this FFQ was the first attempt to create a practical dietary intake instrument targeted at a national level nutrition and health survey. This population-specific FFQ provides a reasonable measure of energy and major nutrient intake in Sri Lankan adults and could be a useful tool to examine the role of diet in the etiology of chronic diseases in this population.

## Additional file

10.1186/s40064-016-1837-x Pearson’s Correlation Coefficients of FFQ 2 and FFQ 1.
