# Supplementary figures and images for: Validity of a food frequency questionnaire to assess nutritional intake among Sri Lankan adults
Source: Springerplus. 2016 Feb 24;5:162. doi: 10.1186/s40064-016-1837-x (PMC4766149; doi:10.1186/s40064-016-1837-x)

Online supplements

Table S1: Pearson’s Correlation Coefficients of FFQ 2 and FFQ 1.


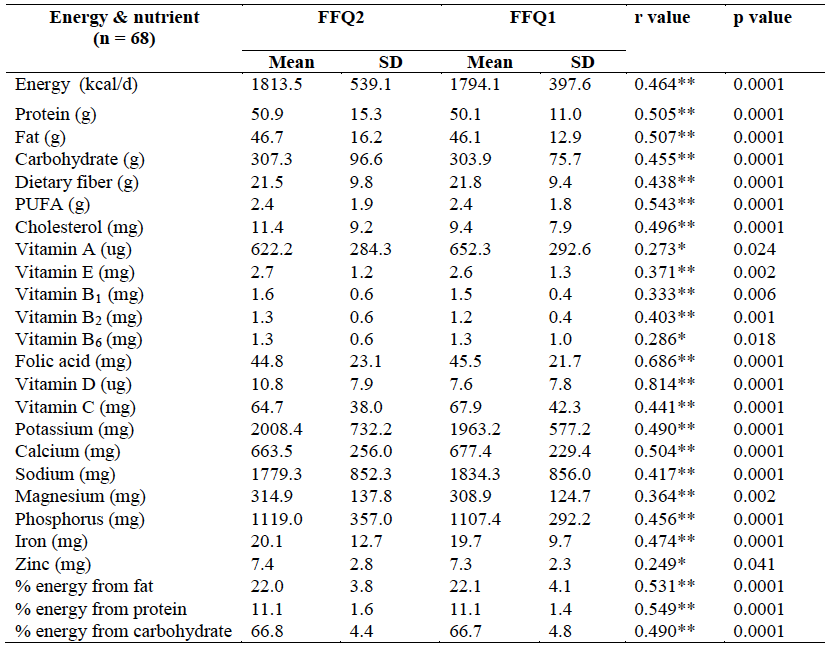

Supplement: Supplementary file 1 — 10.1186/s40064-016-1837-x Pearson’s Correlation Coefficients of FFQ 2 and FFQ 1. [file 40064_2016_1837_MOESM1_ESM.docx]
